# Efficacy and safety of terlipressin infusion during liver surgery: a meta-analysis

**DOI:** 10.1007/s13304-025-02197-y

**Published:** 2025-04-16

**Authors:** Lin Ding, Yi Duan, Zuozhi Li, Qiyue Wu, Lan Yao, Zhifeng Gao

**Affiliations:** 1 Department of Anesthesiology, Beijing Tsinghua Changgung Hospital, School of Clinical Medicine, Tsinghua Medicine, Tsinghua University, No. 168 Litang Road, Beijing, 102218 China; 2 Department of Anesthesiology, Peking University International Hospital, Beijing, China; 3 Department of Cardiology, Fuwai Hospital, National Center for Cardiovascular Disease, Chinese Academy of Medical Sciences and Peking Union Medical College, Beijing, China

**Keywords:** Terlipressin, Liver surgery, Blood loss, Meta-analysis

## Abstract

Although numerous studies have investigated terlipressin (TP) administration in liver surgery to mitigate bleeding, its efficacy remains controversial. This meta-analysis evaluates the effects of TP on estimated blood loss (EBL), blood transfusion requirements, and patient outcomes. We systematically searched PubMed, EMBASE, Cochrane Library, and Web of Science (WOS) for studies on perioperative TP use in liver surgery from their inception through February 2024. Only English-language publications were included. Primary outcomes included EBL and allogeneic blood transfusion volume. Twelve studies involving 988 eligible subjects were included. No significant differences were observed in EBL (weighted mean difference [WMD] =  − 99.09; 95% confidence interval [CI], − 318.41 to 120.24; P = 0.38), red blood cell (RBC) transfusion volume (standardized mean difference [SMD] =  − 0.10; 95% CI =  − 0.74 to 0.54; P = 0.76), or fresh frozen plasma (FFP) transfusion volume (SMD = 0.07; 95% CI =  − 0.24 to 0.37; P = 0.67). Subgroup analysis demonstrated that continuous TP infusion significantly reduced intraoperative EBL (WMD =  − 336.22; 95% CI =  − 562.13 to − 110.31; P = 0.004). TP infusion does not reduce intraoperative EBL or allogeneic blood transfusion requirements in liver surgery. However, continuous TP infusion may lower EBL.

*PROSPERO registration number*: CRD42023450333.

**Supplementary Information:**

The online version contains supplementary material available at https://doi.org/10.1007/s13304-025-02197-y.

## Introduction

Liver diseases account for over 2 million deaths annually, representing 4% of global mortality [1]. In the United States, annual medical costs for patients with chronic liver disease or cirrhosis increased by 4.3% per year from 1996 to 2016 (95% confidence interval [CI], 2.8–5.8%) [2]. For individuals with liver cancer or end-stage liver disease, liver resection or transplantation remains a critical therapeutic option [3, 4].

Bleeding is a common complication of abdominal surgeries, including liver procedures. Intraoperative blood loss during liver resection ranges from 200 to 2000 mL per patient [5], and is strongly associated with the need for allogeneic red blood cell (RBC) transfusion. Perioperative RBC transfusion rates vary by procedure, reaching 50.5–62.6% in liver transplantation and 17–23% in liver resection [6–8]. Both bleeding and allogeneic RBC transfusion are independent risk factors for postoperative complications, impacting short- and long-term outcomes [9]. Elevated portal vein pressure (≥ 20 cmH_2_O) a key prognostic marker for adverse outcomes (hazard ratio [HR] = 1.74; 95% CI 1.24–3.03) [10] and correlates with complications such as hepatic failure, variceal rupture, and coagulation disorders.

Terlipressin (TP), a synthetic antidiuretic hormone analogue, activates V1 receptors in splanchnic artery smooth muscle and V2 receptors in the renal collecting tubule [11, 12]. This induces vasoconstriction in the splanchnic circulation, redirecting blood from viscera to systemic circulation. In liver circulation, TP reduces portal vein blood flow and pressure [13, 14]. By reducing portal vein blood flow and pressure [13, 14], TP may theoretically decrease EBL and transfusion needs during liver surgery [15], while potentially mitigating portal hypertension-related complications.

Recent studies have explored TP use in liver surgery to minimize intraoperative bleeding [16–21, 21–24, 24, 27]; however, its efficacy remains unclear, likely due to heterogeneity in study populations, surgical techniques, TP protocols, and transfusion strategies. Furthermore, limited meta-analyses have evaluated TP’s safety in this context. Notably, TP-induced bradycardia or hypertension may necessitate treatment discontinuation and is a potential risk factor for postoperative cardiac complications [21, 24]. This meta-analysis aims to evaluate TP’s effects on EBL, allogeneic blood transfusion, and patient outcomes following liver surgery.

## Materials and methods

This meta-analysis adhered to Cochrane Systematic Review guidelines, a predefined protocol [25] and the 2020 PRISMA standards [29] (Supplementary Material 1). The protocol was registered with PROSPERO (CRD42023450333) on August 14, 2023 (registration number: CRD42023450333).

### Literature search strategy

A comprehensive search of PubMed, EMBASE, Cochrane Library, and Web of Science (WOS) was conducted to identify studies on perioperative TP use in liver surgery (inception to July 2023), with an updated search in February 2024. We used a combination of subject-specific and free-text terms. The final search strategy was refined through iterative searches. Search terms included *terlipressin* (e.g., Glycylpressin, TGLVP), *liver* (e.g., hepatic), and *surgery* (e.g., surgical procedure). The full strategy is detailed in Supplementary material 2. Two researchers (LD and YD) independently screened studies, resolving discrepancies via consensus.

### Study selection criteria

Included studies met the following criteria: (1) Randomized controlled, case–control, or cohort design; (2) Published by February 2024; (3) Enrolled adults (≥ 18 years) undergoing elective liver surgery, including partial hepatectomy (PH) and orthotopic liver transplantation (OLT); (4) Compared TP with alternative treatments; (5) Reported ≥ 1 efficacy (e.g., EBL, allogeneic blood transfusion, intensive care unit [ICU] stay, length of stay [LOS], intraoperative urine output [UOP], incidence of acute kidney injury [AKI], or postoperative liver and kidney function) or safety outcome (e.g., postoperative complications, TP-related adverse effects).

Exclusion criteria were as follows: (1) Animal studies, case reports, case series, editorials, reviews, letters, conference abstracts, uncontrolled trials, and unpublished or duplicate articles; (2) Pediatric studies; (3) Studies with insufficient data to calculate risk ratio (RR) or weighted mean difference (WMD)/standardized mean difference (SMD); (4) Non-English-language articles; and (5) Studies in which both groups received TP treatment.

### Outcomes

Primary outcomes were EBL, RBC transfusion volume, and fresh frozen plasma (FFP) transfusion volume. Secondary outcomes included Intraoperative urine output (UOP), operating time, ICU stay, LOS, AKI, and postoperative liver/kidney function (peak alanine aminotransferase [ALT], aspartate aminotransferase [AST], and serum creatinine [sCr] levels, and values at the last follow-up). Other secondary outcomes were postoperative complications (pneumonia, reoperation, biliary complications, vascular thrombosis, intra-abdominal hematoma, rejection) and TP-related adverse events (severe bradycardia, new-onset hypertensive crisis, and peripheral skin changes). Definitions for all outcomes are provided in Supplementary material 3.

### Data extraction

Data extraction was independently performed by two investigators (LD and YD) using standard forms. Missing data were obtained by contacting the corresponding author. Disagreements were resolved by a third investigator (ZG).

We extracted the following data from the included studies: first author, publication year, study duration, country, design, sample size, patient age, sex distribution, body mass index (BMI), Model for End-Stage Liver Disease (MELD) score, preoperative ALT, AST, and sCr, follow-up duration, EBL, allogeneic blood transfusion (total blood products and transfusions of RBC, FFP, and platelets), UOP, operating time, postoperative ICU stay, LOS, AKI incidence, liver and kidney function (postoperative ALT, AST, and sCr), postoperative complications (pneumonia, reoperation, biliary complications, vascular thrombosis, intra-abdominal hematoma, rejection), and TP-related adverse events (severe bradycardia, new-onset hypertensive crisis, and peripheral skin color changes). For studies reporting continuous variables as median and range or interquartile range, we calculated the mean ± standard deviation using validated methods [26, 27].

### Quality assessment

We assessed the risk of bias in randomized controlled trials (RCTs) using version 2 of the Cochrane Collaboration’s RoB tool (RoB 2) [28], rather than the original RoB tool outlined in our prior protocol [25], to address its limitations and improve reliability. Although our protocol initially proposed stratifying RCT quality by the Jadad score, we exclusively used RoB 2 due to overlap with the Jadad approach. For case–control studies, we evaluated methodological quality, comparability, and outcomes using the Newcastle–Ottawa Scale (NOS), with scores of 7–9 indicating high quality [29, 30].High-quality studies generally have a lower risk of bias. We assessed evidence levels using the Grading of Recommendations, Development, and Evaluation (GRADE) framework, categorizing outcomes as high, moderate, low, or very low based on study design, risk of bias, inconsistency, indirectness, imprecision, and publication bias [31, 32]. Two investigators (LD and YD) independently evaluated the quality and evidence level of each eligible study, resolving discrepancies through discussion.

### Data synthesis and analysis

Binary categorical variables were expressed as risk ratios (RR), continuous variables as WMD, and SMD for non-unified units. Heterogeneity was assessed using the chi-square test (Cochran's Q) and the I^2^ statistic [33]. A P-value < 0.05 or I^2^ ≥ 50% was considered indicative of substantial heterogeneity. For low heterogeneity (I^2^ < 50%), the Mantel–Haenszel fixed-effects model was applied, while for high heterogeneity (I^2^ ≥ 50%), the Inverse Variance random-effects model was used.

Stratified analyses of primary outcomes were performed using meta-regression based on predefined criteria [25]: (1) study location, (2) sample size, (3) risk of bias, (4) follow-up duration, (5) control group intervention, (6) TP infusion mode, (7) surgery type, and (8) study design. Meta-regression was applied to EBL when at least five studies were included. Excluded criteria included sample size (only one study with > 100 patients), follow-up duration (unclear in most studies), intervention type (one study did not specify the control agent), and study design (all studies were RCTs). Surgery type and TP infusion mode were selected for meta-regression based on clinical relevance.

We also conducted a leave-one-out sensitivity analysis to evaluate the influence of each included study on the combined outcome for primary measures with significant heterogeneity.

We assessed publication bias visually using Egger's regression test [34] in Stata V.12.0 (Stata Corp, College Station, TX, USA) for outcomes with three or more studies.

Data analyses were conducted using Review Manager V.5.3 and Stata V.12.0, with a 95% CI reported for all measures. A P-value < 0.05 was considered statistically significant.

## Results

### Literature search and study characteristics

**Fig.1:**
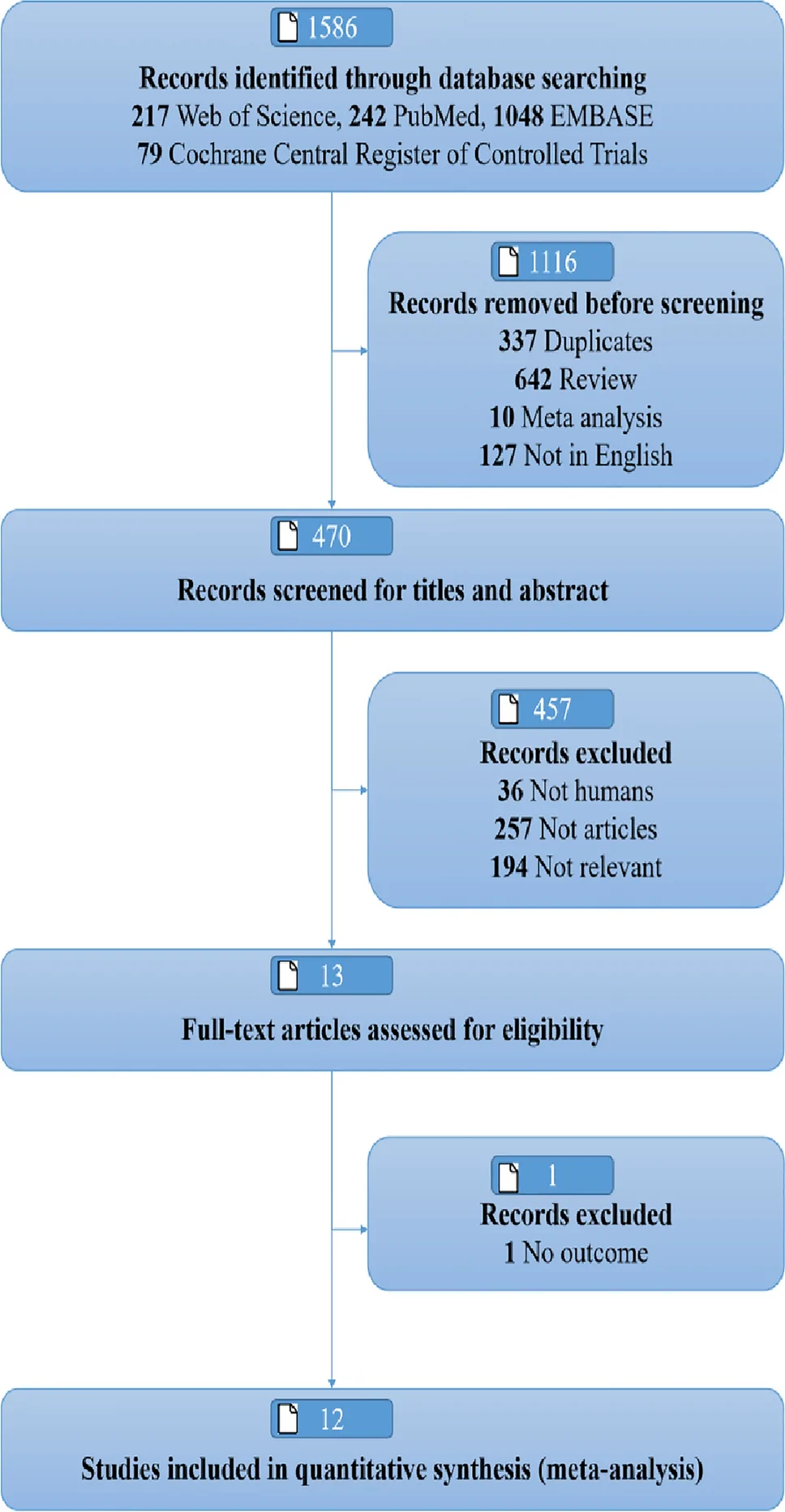
Study selection flowchart illustrating literature screening and inclusion process

A total of 1,586 articles were initially identified through database searches. After removing duplicates, reviews, meta-analyses, and non-English publications, 470 records underwent title/abstract screening. Ultimately, 12 studies involving 988 patients (448 TP, 540 controls) met the inclusion criteria (Fig. 1). Ten studies were randomized controlled trials (RCTs) [16–24, 27], and two were retrospective case–control studies [21, 24]. Nine studies utilized continuous TP infusion, while three employed intermittent administration. Baseline characteristics are summarized in Table 1 and quality assessments are provided in Supplementary materials 4 and 5.

**Table 1:** Baseline characteristics of included studies

Year, First authors	Study period	Country	Study design	Patients(n) TP/without TP	Median Follow-up (days)	Inclusion criteria	Exclusion criteria	TP dose/administration mode	Comparison	Outcomes
2019 Abbas [16]	2015.9–2016.12	Egypt	prospective	42/42	Not specified	Patients ≥ 18 years old, ASA I- II, and elective resection of two or more liver segments	CTP B or C, clinically significant portal hypertension (splenomegaly, thrombocytopenia with platelets < 10^11^/L, oesophageal varices grade 2 or more), preoperative renal failure (GFR < 50 mL/min), heart failure, Brady arrhythmia (heart rate < 60/min), history of haemorrhagic stroke, uncontrolled arterial hypertension (BP > 160/100 mmHg) and pregnancy	Bolus(1 mg over 30 min) after induction of anaesthesia, then a continuous infusion(2 ug/kg/h) until 4 h postoperatively	Saline	The primary outcome was the amount of intraoperative blood loss. Secondary outcomes included the number of patients need blood transfusion, the number of red blood cell units transfused, hospital stay, ICU stay, the incidence of surgical site infection or pneumonia and the incidence of intra-abdominal haematoma
2013 Fayed [17]	2011.1–2012.7	Egypt	prospective	40/40	more than 7 days	Portal vein with a straight section for at least 3 cm, angles between the Doppler beam and the longitudinal axis of the portal vein and the right hepatic artery less than 60°, and high-quality Doppler signals of the right hepatic artery and the interlobar artery of the kidney	History of angina or myocardial infarction, evidence of cardiac decompensation, severe hemodynamic instability, renal disease (proteinuria and ultrasonographic evidence of obstructive uropathy or parynchemal renal disease), and age less than 18 years	3 ug/kg/h at the beginning of surgery, then 1.5 ug/kg/h after reperfusion and continued for 3 postoperative days	Nor-adrenaline	EBL, UOP, liver function tests(e.g., AST,ALT), sCr
2023 Hashim [18]	2019–2023	Egypt	prospective	15/15	Not specified	Portal hypertension (portal pressure > 20 mmHg or hepatic venous pressure gradient > 5 mmHg), along with chronic liver disease and scheduled as recepients for LDLT	Age < 21 years, history of myocardial infarction or angina, cardiac decompensation, arrhythmias, uncontrolled hypertension, parenchymal renal disease (proteinuria, creatinine > 3 mg/dl, GFR < 60 ml/min by isotope scanning of the kidney) or obstructive uropathy, retransplantation, and those with hepatic encephalopathy and cerebrovascular diseases	Bolus (1 mg over 10 min, every 4 h) after portal vein exposure	Nor-epinephrine	Primary outcome was effect of intraoperative terlipressin on hepatic hemodynamics (portal venous pressure, portal venous flow and hepatic artery resistive index). Secondary outcome was effect of intraoperative terlipressin on systemic hemodynamics (BP, heart rate, UOP and the amount of transfused blood products and doses of norepinephrine supplementation)
2012 Hong [19]	2009.6–2011.3	Republic of Korea	prospective	21/20	10 days after surgery	Patients undergoing LDLT	SVRI ≥ 1700 dyne·s/cm^5^/m^2^, PVRI ≥ 150dyne·s/cm^5^/m^2^, history of angina or myocardial infarction, evidence of cardiac decompensation, severe haemodynamic instability, primary renal dysfunction, retransplantation, and age of < 18 years	Continuous infusion(1.0–4.0 ug/kg/h) until 60 min after reperfusion	Conventional inotropic and vasopressor agents	Haemodynamic variables, global tissue oxygenation variables, acid–base balance variables (arterial pH and lactate), the amount of bleeding, fluid requirement, transfusion volume and hourly UOP, the incidence of rejection
2015 Ibrahim [20]	2013	Egypt	prospective	25/25	4 days after surgery	Male and female patients aged 18–55 years with model of end stage liver disease score between 12 and 20 undergoing LDLT, portal vein after transplantation of adequate length for the Doppler beam to allow for high-quality signals	SVRI ≥ 1700 dyne·s/cm^5^/m^2^, history of myocardial infarction, renal dysfunction, severe oesophageal varices and irregular heart rhythm	1.0–4.0 ug/kg/h at the beginning of surgery till day 4	Placebo of crystalloids	UOP, conventional renal and liver function laboratory and blood concentration of lactate
2019 Karaaslan [21]	2014.4–2016.7	Turkey	retrospective	48/38	Not specified	Adult patients who received LDLT, due to end-stage hepatic disease	Patients younger than 18 years old, retransplantation, patients with hepatopulmonary syndrome, and patients who had creatinine levels above 1.2 mg/dL	3 ug/kg/h after induction of anaesthesia, then then 1.5 ug/kg/h after arterial anastomosis and continued for the subsequent 3 days	Patients who not received TP	Renal functions, hepatic functions, and complications
2020 Kohler [21]	2013.11–2017.12	Switzerland	prospective	65/64	42 days	Expected resection of at least three liver segments, age above 18 years	preoperative severe renal failure (estimated glomerular filtration rate (eGFR) < 30 ml/min), severe liver dysfunction (CTP grade C), hyponatremia (< 132 mmol/L), severe aortic or mitral regurgitation, symptomatic coronary heart disease, bradycardic arrhythmia, peripheral arterial occlusive disease (Fontaine stage II), dilatative arteriopathy, history of subarachnoidal bleeding, decompensated hypertension, mesenteric ischemia, septic shock and pregnancy	Bolus(1 mg over 2 h, every 6 h) prior to mobilization of the liver for 5 days	Saline	Blood loss, operating time, liver and kidney function parameters, AKI, postoperative complications, and adverse drug reactions to TP
2019 Mahdy [22]	2016.4–2017.7	Egypt	prospective	25/25	Not specified	Adult patients aged > 18 years old with ASA I-II undergoing major elective hepatobiliary surgery	Preoperative renal failure, severe liver dysfunction (CTP grade C), hyponatremia (Na + < 132 mmol/L), severe valvular heart disease, heart failure, symptomatic coronary heart disease, bradycardic arrhythmia (heart rate < 60/min), peripheral artery occlusive disease (clinical stadium II-IV), uncontrolled arterial hypertension (BP > 160/100 mmHg despite intensive treatment), pregnancy and intraoperative need for Pringle maneuver	Bolus(1 mg over 30 min) after exposure of the portal vein, then a continuous infusion(2 ug/kg/h) until 4 h postoperatively	Saline	Packed red blood cells, FFP, UOP, blood loss, operating time, ICU stay, conventional renal function and AKI incidence
2015 Mukhtar [24]	2006.1–2013.6	Egypt	retrospective	99/204	Not specified	Patients with Child–Pugh C cirrhosis undergoing LDLT	Patients aged < 18 years, those with portal vein thrombosis, and those with dialysis before transplantation	Bolus(1 mg over 30 min) after induction of anaesthesia, then a continuous infusion(2 ug/kg/h) for 2 days	Patients who not received TP	Vascular or biliary complications, the incidence of acute rejection and reoperation, ICU stay, LOS, and AKI
2011 Mukhtar [23]	2008.6–2010.1	Egypt	prospective	15/15	Not specified	Patients with end-stage liver disease undergoing LDLT: the presence of CTP grade C liver cirrhosis; and significant portal hypertension (portal pressure > 20 mmHg)	Age < 18 yrs, retransplantation, a history of upper abdominal surgery, portal vein thrombosis (diagnosed using preoperative duplex ultrasound), and primary renal dysfunction	Bolus(1 mg over 30 min) after exposure of the portal vein, then a continuous infusion(2 ug/kg/h) for 2 days	Saline	Renal Function, transfusions of packed red blood cells and FFP, UOP, liver function tests, ICU stay, and LOS
2017 Reddy [24]	2012.7–2013.12	India	prospective	21/20	More than 35 days	Adults (age > 18 years) undergoing their first LDLT	Patients undergoing liver transplantation for acute liver failure, multiorgan transplants, high risk of PTRD (sCr > 1.5 mg/dL at time of transplant), and significant cardiac morbidity (coronary artery disease or cirrhotic cardiomyopathy)	Bolus(1 mg over 30 min), then a continuous infusion(2 ug/kg/h) for 72 h	Saline	EBL, UOP, intraoperative red cell transfusion, peak AST, the incidence of reoperation and AKI, LOS, ICU stay, TP-related adverse effects
2023 Zhang [27]	2019.3–2021.1	China	prospective	32/32	Not specified	Adult patients (≥ 18 years) who were scheduled for LT with a whole liver graft procured from deceased donors	Any history of allergy to vasopressin or its analogs, severe cardiopulmonary disease (e.g., angina, myocardial infarction, heart failure, moderate-to-severe pulmonary hypertension, or cor pulmonale), primary kidney disease (e.g., nephritis, azotemia, or renal failure), septic shock, preoperative vasopressor support, and refusal of informed consent	1 mg after portal vein clamping	Placebo	EBL, RBC and FFP transfusion, operating time, the incidence of reoperation and AKI, postoperative hepatic and renal function, LOS, ICU stay

TP, terlipressin; ASA, American Society of Anesthesiologists; CTP, Child—Turcotte—Pugh; GFR, glomerular filtration rate; BP, blood pressure; ICU, intensive care unit; EBL, estimated blood loss; UOP, urine output; AST, aspartate aminotransferase; ALT, alanine aminotransferase; sCr, serum creatinine; LDLT, living donor liver transplantation; SVRI, systemic vascular resistance index; PVRI, pulmonary vascular resistance index; eGFR, estimated glomerular filtration rate; AKI, acute kidney injury; FFP, fresh frozen plasma; LOS, length of stay, PTRD, post-transplant renal dysfunction; LT, liver transplantation

### Demographic characteristics

No significant differences were observed between groups in age (WMD = 0.96; 95% CI =  − 1.05 to 1.18; P = 0.35), gender distribution (female/total, RR = 0.93; 95% CI = 0.73 to 1.18; P = 0.54), MELD score (WMD = 0.19; 95% CI =  − 0.38 to 0.77; P = 0.51), preoperative AST (WMD =  − 0.45; 95% CI =  − 4.77 to 0.38; P = 0.84), ALT (WMD = 1.38; 95% CI =  − 4.40 to 7.16; P = 0.64), and sCr (WMD = 0.03; 95% CI =  − 0.02 to 0.08; P = 0.22). However, body mass index (BMI) differed significantly (WMD = 0.57; 95% CI = 0.03 to 1.12; P = 0.04) (Supplementary material 6).

### Primary outcomes

#### EBL

**Fig.2:**
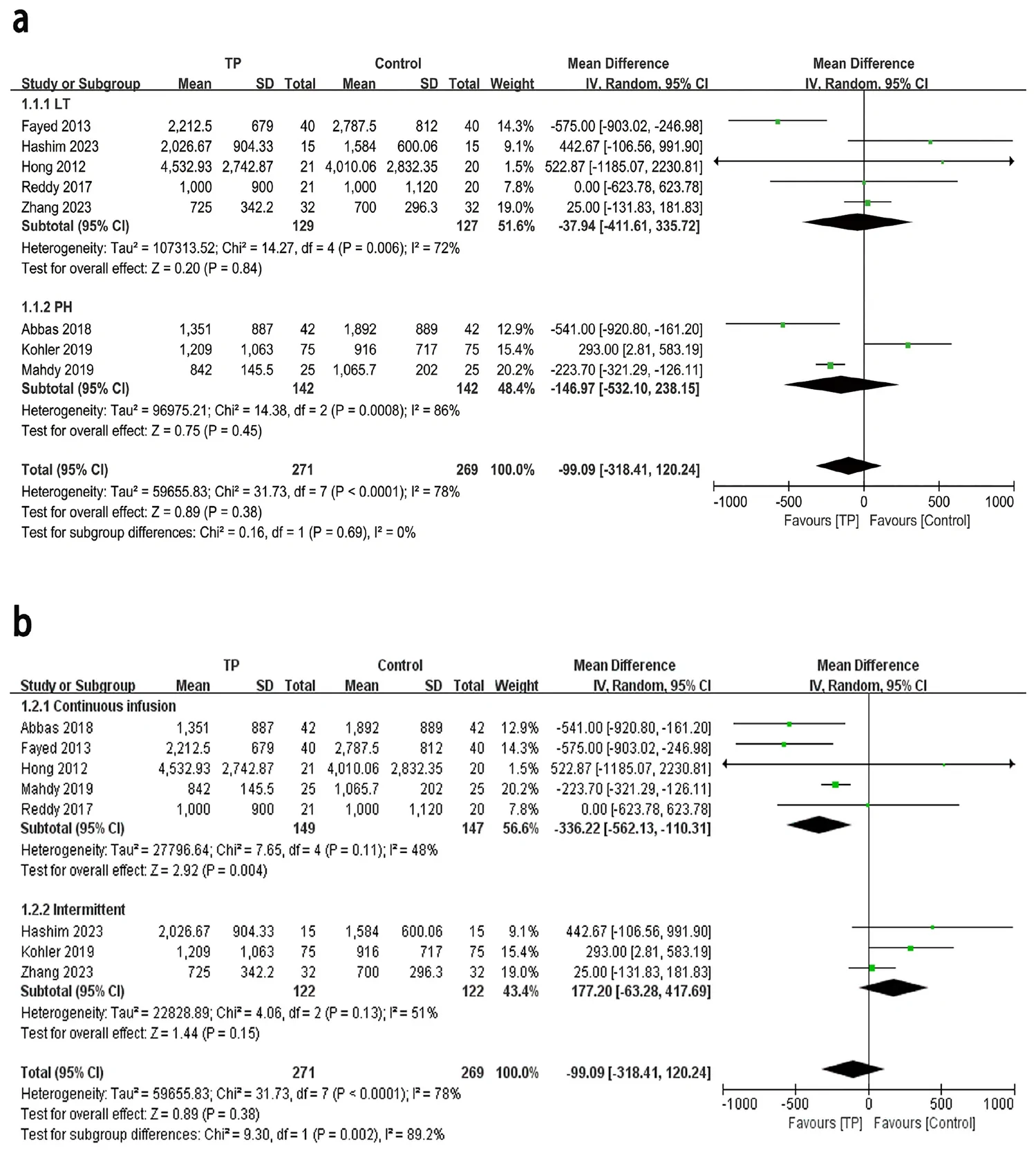
Forest plot of estimated blood loss (EBL) based on (**a**) the type of surgery and (**b**) the mode of TP infusion between TP and control groups

EBL was analyzed in eight studies with 540 patients (271 TP, 269 no TP) [16–19, 21, 22, 24, 27]. Pooled analysis revealed no statistically significant difference in EBL between the groups (WMD =  − 99.09; 95% CI =  − 318.41 to 120.24; P = 0.38), though substantial heterogeneity was observed (I^2^ = 78%, P < 0.0001). Meta-regression analysis identified TP infusion modality as a significant factor (P = 0.020), whereas surgical approach showed no significant association (P = 0.702). Subgroup analysis by surgery type demonstrated comparable EBL values between the LT and PH subgroups (P = 0.69, Fig. 2a). Subgroup analysis stratified by TP infusion method showed significantly reduced EBL with continuous infusion (WMD =  − 336.22; 95% CI =  − 562.13 to − 110.31; P = 0.004), in contrast to intermittent infusion regimens which showed no intergroup difference (WMD = 177.20; 95% CI =  − 63.28 to 417.69; P = 0.15). Notably, the between-subgroup difference reached statistical significance (P = 0.002) (Fig. 2b).

#### RBC transfusion volume

Four studies comprising 142 patients (72 TP, 70 no TP) reported RBC transfusion volumes [18, 19, 23, 24]. The pooled analysis demonstrated comparable transfusion requirements between groups (SMD =  − 0.10; 95% CI =  − 0.74 to 0.54; P = 0.76), albeit with substantial between-study heterogeneity (I^2^ = 72%, P = 0.01) (Supplementary material 7).

#### FFP transfusion volume

FFP transfusion data were available from four trials including 165 patients (83 TP, 82 no TP) [18, 19, 23, 27]. No significant intergroup differences were detected in FFP utilization (SMD = 0.07; 95% CI =  − 0.24 to 0.37; P = 0.67), with minimal observed heterogeneity across studies (I^2^ = 0%, P = 0.67) (Supplementary material 8).

### Secondary outcomes

#### AKI rate

**Fig.3:**
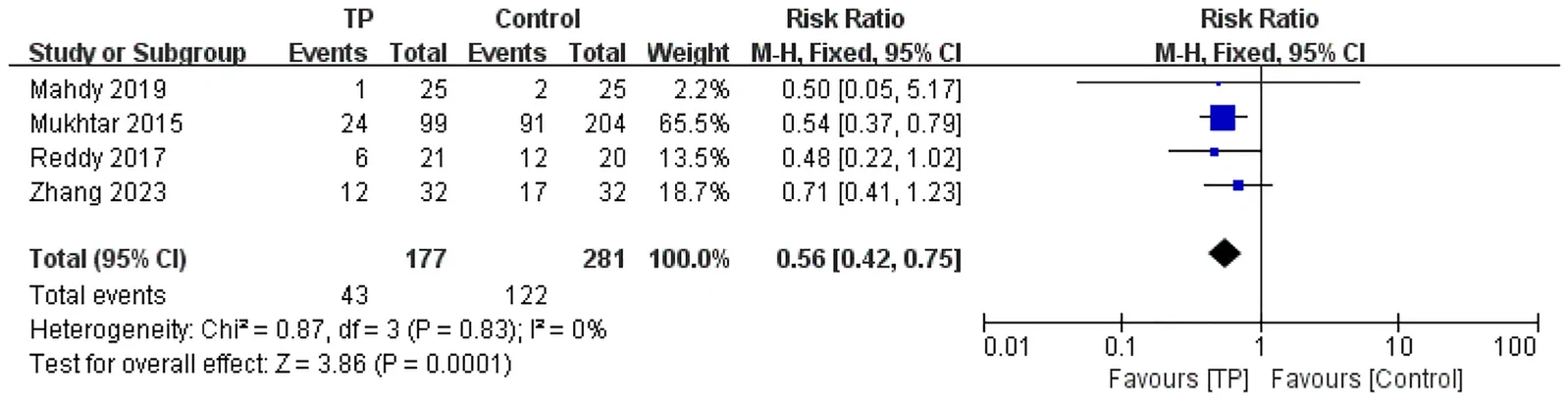
Forest plot of acute kidney injury (AKI) incidence between TP and control groups. LT, liver transplantation; PH, partial hepatectomy

Four clinical trials encompassing 458 patients (177 TP, 281 non-TP) evaluated AKI occurrence [22, 24, 24, 27]. The TP group demonstrated a significantly reduced risk of AKI (RR = 0.56; 95% CI = 0.42–0.75; P = 0.0001), with no significant heterogeneity across studies (I^2^ = 0%, P = 0.83) (Fig. 3).

#### Intraoperative UOP

Intraoperative UOP data were extracted from six studies involving 306 patients (154 TP, 152 non-TP) [17, 19, 20, 23, 24, 27]. Pooled analysis demonstrated significantly higher intraoperative UOP in the TP group (SMD = 0.48; 95% CI = 0.06 to 0.89; P = 0.02), notwithstanding significant between-study heterogeneity (I^2^ = 67%, P = 0.009) (Supplementary material 9).

#### Peak sCr and sCr at last follow-up

Peak sCr was assessed in seven studies comprising 453 patients (231 TP, 222 no TP) [16, 17, 19–22, 27]. The TP group exhibited statistically lower peak sCr values compared to controls (WMD =  − 0.13; 95% CI =  − 0.26 to − 0.00; P = 0.005), though moderate heterogeneity was observed (I^2^ = 52%, P = 0.05) (Supplementary material 10a).

Final follow-up sCr data from five studies (176 TP, 169 non-TP) [16, 17, 20–22] showed no significant intergroup differences (WMD =  − 0.15; 95% CI =  − 0.37–0.08; P = 0.21), despite pronounced heterogeneity (I^2^ = 85%, P < 0.0001) (Supplementary Material 10b).

#### Postoperative hepatic function

Pooled analyses of peak and final follow-up AST/ALT revealed no significant differences between groups (Supplementary Material 11).

#### LOS

**Fig.4:**
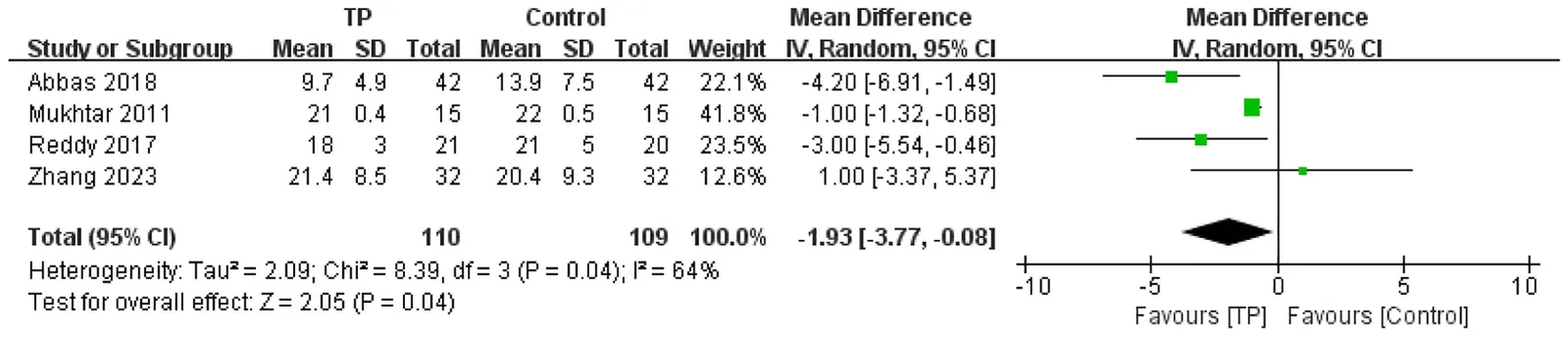
Forest plot of hospital length of stay (LOS) between TP and control groups

Four studies involving 219 patients (110 TP, 109 non-TP) evaluated hospitalization duration [16, 23, 24, 27]. Meta-analysis indicated significantly prolonged LOS in the control group (WMD =  − 1.93; 95% CI =  − 3.77 – − 0.08; P = 0.04), with moderate heterogeneity across studies (I^2^ = 64%, P = 0.04) (Fig. 4).

#### TP-related adverse effects

Two trials (86 TP, 84 controls) reported TP-related complications [21, 24]. No significant between-group differences were observed for severe bradycardia (RR = 3.70; 95% CI = 0.94 to 14.51; P = 0.06; Supplementary Material 12a), new-onset hypertensive crisis (RR = 1.70; 95% CI = 0.76 to 3.82; P = 0.20; Supplementary Material 12b), and peripheral skin discoloration (RR = 0.32; 95% CI = 0.03 to 3.03; P = 0.32; Supplementary Material 12c). No significant heterogeneity was detected.

#### Additional secondary outcomes

Pooled analyses of operative duration (Supplementary material 13), ICU stay (Supplementary material 14), and postoperative complications (Supplementary material 15) showed no statistically significant intergroup disparities.

### Sensitivity analysis and publication bias

Leave-one-out sensitivity analyses for EBL and RBC transfusion volume confirmed the robustness of pooled estimates, with no single study significantly altering the WMD/SMD significance (Supplementary material 16a, b). Egger's regression tests demonstrated no evidence of publication bias (Supplementary material 17).

### GRADE evidence quality assessment

Using the GRADE framework, the quality of evidence for all outcomes ranged from moderate to very low certainty (Table 2).

**Table 2:** GRADE classification of quality of evidence

Outcome	No. of studies	RCT	Cohort	Case–control/cross-sectional	Risk of bias	Inconsistency	Indirectness	Imprecision	Publication bias	Plausible confounding	Magnitude of effect	Dose–response gradient	Quality
EBL	8	8	0	0	No serious Risk	Serious Inconsistency	No serious Indirectness	Serious Imprecision	Na	Would not Reduce effect	no	no	Low
RBC transfusion volume	4	4	0	0	Serious risk	Serious Inconsistency	No serious Indirectness	Serious Imprecision	Na	Would not Reduce effect	no	no	Very low
FFP transfusion volume	4	4	0	0	Serious risk	No serious Inconsistency	No serious Indirectness	Serious Imprecision	Na	Would not Reduce effect	no	no	Low
Operating time	5	5	0	0	No serious Risk	No serious Inconsistency	No serious Indirectness	Serious Imprecision	Na	Would not Reduce effect	no	no	Moderate
Intraoperative UOP	6	6	0	0	Serious risk	Serious Inconsistency	No serious Indirectness	Serious Imprecision	Na	Would not Reduce effect	no	no	Very low
ICU stay	5	5	0	0	No serious Risk	Serious Inconsistency	No serious Indirectness	Serious Imprecision	Na	Would not Reduce effect	no	no	Low
LOS	4	4	0	0	No serious Risk	Serious Inconsistency	No serious Indirectness	Serious Imprecision	Na	Would reduce Effect	no	no	Moderate
Hepatic function during follow-up													
AST at last follow-up	4	3	0	1	Serious risk	No serious Inconsistency	No serious Indirectness	Serious Imprecision	Na	Would not Reduce effect	No	No	Low
Peak AST	5	4	0	1	No serious Risk	Serious Inconsistency	No serious Indirectness	Serious Imprecision	Na	Would not Reduce effect	No	No	Low
ALT at last follow-up	5	4	0	1	Serious risk	No serious Inconsistency	No serious Indirectness	Serious Imprecision	Na	Would not Reduce effect	No	No	Low
Peak ALT	5	4	0	1	No serious Risk	Serious Inconsistency	No serious Indirectness	Serious Imprecision	Na	Would not Reduce effect	No	No	Low
Renal function during follow-up													
AKI rate	4	3	0	1	No serious Risk	No serious Inconsistency	No serious Indirectness	Serious Imprecision	Na	Would not Reduce effect	No	No	Moderate
sCr at last follow-up	5	4	0	1	No serious Risk	Serious Inconsistency	No serious Indirectness	Serious Imprecision	NA	Would not Reduce effect	No	No	Low
Peak sCr	7	6	0	1	No serious Risk	No serious Inconsistency	No serious Indirectness	Serious Imprecision	NA	Would not Reduce effect	No	No	Moderate
Postoperative complications													
Pneumonia	3	2	0	1	No serious Risk	No serious Inconsistency	No serious Indirectness	Serious Imprecision	NA	Would not Reduce effect	No	No	Moderate
Reoperation	4	2	0	2	No serious Risk	No serious Inconsistency	No serious Indirectness	Serious Imprecision	NA	Would not Reduce effect	No	No	Moderate
Biliary complications	3	1	0	2	Serious risk	No serious Inconsistency	No serious Indirectness	Serious Imprecision	NA	Would not Reduce effect	No	No	Low
Vascular complications	3	1	0	2	Serious risk	No serious Inconsistency	No serious Indirectness	Serious Imprecision	NA	Would not Reduce effect	No	No	Low
Intra-abdominal haematoma	2	2	0	0	No serious Risk	No serious Inconsistency	No serious Indirectness	Serious Imprecision	NA	Would not Reduce effect	No	No	Moderate
Rejection	2	1	0	1	Serious risk	No serious Inconsistency	No serious Indirectness	Serious Imprecision	NA	Would not Reduce effect	No	No	Low
Tp-related adverse effects													
Severe bradycardia	2	2	0	0	No serious Risk	No serious Inconsistency	No serious Indirectness	Serious Imprecision	NA	Would not Reduce effect	No	No	Moderate
New-onset hypertension crisis	2	2	0	0	No serious Risk	No serious Inconsistency	No serious Indirectness	Serious Imprecision	NA	Would not Reduce effect	No	No	Moderate
Peripheral skin changes	2	2	0	0	No serious Risk	No serious Inconsistency	No serious Indirectness	Serious Imprecision	NA	Would not Reduce effect	No	No	Moderate

GRADE, Grades of Recommendation, Assessment, Development, and Evaluation; EBL, estimated blood loss; RCT, randomized controlled trial; NA, not applicable; RBC, red blood cell; FFP, fresh frozen plasma; UOP, urine output; ICU, intensive care unit; LOS, length of stay; AST, aspartate aminotransferase; ALT, alanine aminotransferase; AKI, acute kidney injury; sCr, serum creatinine; TP, terlipressin

## Discussion

Multiple clinical strategies are implemented to mitigate intraoperative blood loss in hepatic surgery [5, 35, 36], encompassing refined surgical techniques (e.g., hepatic blood flow occlusion), pharmacological interventions, and hemodynamic optimization protocols. Nevertheless, robust evidence validating the efficacy of pharmacological interventions for blood loss reduction remains scarce [37]. TP, a synthetic analogue of antidiuretic hormone, has emerged as a potential adjunct in this context. Although TP is primarily indicated for hepatorenal syndrome (HRS) and acute variceal hemorrhage (AVB) [38, 39, 44], current guidelines provide no recommendations for its perioperative use. Initial clinical investigations by Mukhtar et al. (2011) in living donor liver transplantation established foundational insights into TP's hemodynamic and renal effects [21]. The efficacy of TP in reducing EBL remains debated. While its safety in cirrhotic populations is well-characterized [40, 46], no meta-analysis has yet assessed its safety in liver surgery. Our meta-analysis of 12 studies (n = 988) systematically assessed TP's therapeutic efficacy and safety in hepatic surgeries, yielding several critical observations.

Recent pharmacological investigations [40] demonstrate that continuous low-dose TP regimens (4 mg/24 h over 5 days) significantly reduce portal vein pressure, control bleeding, and maintaining favorable safety outcomes in cirrhotic patients with AVB, supporting its effectiveness in reducing EBL and the need for allogeneic blood transfusion. Our pooled analysis showed no significant difference between TP and control groups. Notably, subgroup analysis identified a clinically relevant 330 ml blood loss reduction with continuous TP infusion. The pharmacokinetics and pharmacodynamics of TP indicate that its active metabolites are released gradually after intravenous administration, maintaining efficacy for 4–6 h and allowing for intermittent administration [41, 42]. A single intravenous dose of 1 mg of TP reduces portal vein pressure within 3 h [43], whereas continuous infusion sustains efficacy throughout. Thus, continuous TP infusion may reduce intraoperative EBL by lowering portal vein pressure. However, continuous infusion of TP did not reduce subsequent allogeneic transfusions, which may be due to differences in transfusion strategies, including different target hemoglobin levels (7 g/dL, 8 g/dL, or 10 g/dL) and the lack of standardized protocols, which may have influenced the results. Some studies used vasoactive agents, such as norepinephrine, in the control group [17, 18]. Norepinephrine, an alpha-adrenergic agonist, reduces portal vein pressure by constricting splanchnic vessels, thereby reducing the effect of TP infusion on allogeneic blood transfusion. Risk factors for blood transfusion include preoperative hemoglobin, comorbidities, disease severity, and intraoperative blood loss, with estimated blood loss being an independent risk factor [44, 45]. Liver surgery carries a high risk of bleeding. Continuous TP infusion in high-risk patients may reduce intraoperative EBL and the need for allogeneic transfusion, thereby reducing the associated adverse outcomes.

Second, TP does not impair postoperative liver function. The observed reductions in AKI incidence and peak serum creatinine levels likely reflect TP's vasomodulatory action. TP targets V1 receptors, induces splanchnic arteriole contraction and redirects blood to the systemic circulation, thereby increasing effective circulating blood volume and MAP. Additionally, TP reduces renal artery resistance and improves renal perfusion pressure to maintain renal function[13]. Four studies evaluating AKI have different distinct diagnostic criteria: AKIN [20, 21], RIFLE [22], and KDIGO [24]. The KDIGO guidelines [46] combine creatinine and urine volume monitoring to categorize AKI into three phases, which facilitates earlier diagnosis, improves detection rates, and reduces mortality. The use of the KDIGO criteria to recalculate the incidence of AKI in the relevant studies was limited by the lack of standardized diagnostic criteria and incomplete data. There was no significant difference in sCr between the two groups at final follow-up, which may be related to the short follow-up period. A multi-center study involving 203 patients with HRS-AKI [47] showed that the median TP response time was 8 days, whereas the median follow-up time in this study was 4 days (range 3–7 days). The long-term protective effect of TP on renal function during liver surgery remains uncertain due to incomplete data, which hinders pooled analysis of changes in renal function and glomerular filtration rate (GFR), a crucial indicator.

Third, TP's association with reduced hospitalization duration may stem from its role in reducing EBL during infusion and protecting renal function. Reduced blood loss reduces the risk of complications related to anemia and organ hypoperfusion and shortens hospital stay [48].

Finally, common adverse effects of TP include bradycardia, finger pallor, abdominal spasm, and diarrhea [49]. Severe bradycardia or hypertension requiring medical intervention are significant adverse reactions. This study found that neither continuous nor intermittent infusion exacerbated the risk of cardiovascular or other complications. However, safety assessment of perioperative use of TP remains limited by small sample sizes and inadequate power to detect rare events.

Our study has several limitations. First, the inclusion of both RCTs and case–control studies introduced limitations related to heterogeneity of study designs. This may affect the consistency and reliability of the study results. Future research specifically investigating separate meta-analyses for each study type is warranted, thereby yielding more robust and interpretable results. Second, the small number of studies and sample sizes limited our ability to control for confounding factors, such as variability in control group interventions and the lack of standardization of TP dose and administration across studies. This inconsistency may lead to variations in treatment effects and complicates the interpretation of the overall results. Third, significant heterogeneity was observed. Although subgroup and sensitivity analyses were performed to assess the stability of the results, the source of heterogeneity for some outcomes remains unclear. Finally, incomplete data prevented us from standardizing AKI diagnostic criteria or assessing long-term outcomes, including in-hospital mortality and survival rates. Also, the length of follow-up in the included studies varied, making it difficult to assess the long-term effects of TP.

In summary, there is limited evidence regarding the efficacy and safety of TP in reducing blood loss and allogeneic transfusion in liver surgery. Well-designed, large-scale randomized studies are needed to evaluate prognosis. These studies will deepen our understanding of the efficacy and safety of TP and help make evidence-based decisions to minimize blood loss and transfusion requirements.

## Conclusion

Current evidence suggests that TP infusion does not reduce EBL during liver surgery. However, continuous TP infusion demonstrates potential efficacy in decreasing intraoperative blood loss, though this effect does not translate into reduced requirements for allogeneic blood transfusion. In high-risk patients undergoing hepatic procedures, continuous intraoperative TP infusion may offer clinical benefits by minimizing surgical hemorrhage and transfusion demands. Given the observed heterogeneity across studies and potential methodological biases, TP administration should be cautiously individualized with rigorous monitoring for adverse events.

## Supplementary Information

Below is the link to the electronic supplementary material.

## Data Availability

The study is based on data from previously published studies and is available online to all.
