# Supplementary material for: Efficacy and safety of terlipressin infusion during liver surgery: a meta-analysis
Source: Updates Surg. 2025 Apr 16;78(5):2063–79. doi: 10.1007/s13304-025-02197-y (PMC13538207; doi:10.1007/s13304-025-02197-y)
Supplement: Supplementary file 1 [file 13304_2025_2197_MOESM1_ESM.docx]

**Supplementary Materials**

| Supplementary Material 1 | PRISMA_2020_Checklist |
| --- | --- |
| Supplementary Material 2 | Detailed search strategy in four databases |
| Supplementary Material 3 | Definition of outcomes in included studies |
| Supplementary Material 4 | Quality evaluation of the eligible RCTs with RoB2 |
| Supplementary Material 5 | Quality evaluation of the eligible case-control studies with NOS |
| Supplementary Material 6 | Demographics of included studies |
| Supplementary Material 7 | Forest plot of red blood cell transfusion between the TP group and the Control group |
| Supplementary Material 8 | Forest plot of fresh frozen plasma between the TP group and the Control group |
| Supplementary Material 9 | Forest plot of intraoperative urine output between the TP group and the Control group |
| Supplementary Material 10 | Forest plot of postoperative serum creatine between the TP group and the Control group |
| Supplementary Material 11 | Forest plot of postoperative hepatic function between the TP group and the Control group |
| Supplementary Material 12 | Forest plot of TP-related adverse events between the TP group and the Control group |
| Supplementary Material 13 | Forest plot of operating time between the TP group and the Control group |
| Supplementary Material 14 | Forest plot of intensive care unit stay between the TP group and the Control group |
| Supplementary Material 15 | Forest plot of postoperative complications between the TP group and the Control group |
| Supplementary Material 16 | Sensitivity analysis |
| Supplementary Material 17 | Egger's regression tests |

**Supplementary Material 1 PRISMA_2020_Checklist**

| **Section and Topic** | **Item #** | **Checklist item** | **Location where item is reported** |
| --- | --- | --- | --- |
| **TITLE** | | |  |
| Title | 1 | Identify the report as a systematic review. | 1 |
| **ABSTRACT** | | |  |
| Abstract | 2 | See the PRISMA 2020 for Abstracts checklist. | 2-3 |
| **INTRODUCTION** | | |  |
| Rationale | 3 | Describe the rationale for the review in the context of existing knowledge. | 4-5 |
| Objectives | 4 | Provide an explicit statement of the objective(s) or question(s) the review addresses. | 5 |
| **METHODS** | | |  |
| Eligibility criteria | 5 | Specify the inclusion and exclusion criteria for the review and how studies were grouped for the syntheses. | 6-7 |
| Information sources | 6 | Specify all databases, registers, websites, organisations, reference lists and other sources searched or consulted to identify studies. Specify the date when each source was last searched or consulted. | 6 |
| Search strategy | 7 | Present the full search strategies for all databases, registers and websites, including any filters and limits used. | 6 |
| Selection process | 8 | Specify the methods used to decide whether a study met the inclusion criteria of the review, including how many reviewers screened each record and each report retrieved, whether they worked independently, and if applicable, details of automation tools used in the process. | 6 |
| Data collection process | 9 | Specify the methods used to collect data from reports, including how many reviewers collected data from each report, whether they worked independently, any processes for obtaining or confirming data from study investigators, and if applicable, details of automation tools used in the process. | 8 |
| Data items | 10a | List and define all outcomes for which data were sought. Specify whether all results that were compatible with each outcome domain in each study were sought (e.g. for all measures, time points, analyses), and if not, the methods used to decide which results to collect. | 7 |
|  | 10b | List and define all other variables for which data were sought (e.g. participant and intervention characteristics, funding sources). Describe any assumptions made about any missing or unclear information. | 8 |
| Study risk of bias assessment | 11 | Specify the methods used to assess risk of bias in the included studies, including details of the tool(s) used, how many reviewers assessed each study and whether they worked independently, and if applicable, details of automation tools used in the process. | 8-9 |
| Effect measures | 12 | Specify for each outcome the effect measure(s) (e.g. risk ratio, mean difference) used in the synthesis or presentation of results. | 9 |
| Synthesis methods | 13a | Describe the processes used to decide which studies were eligible for each synthesis (e.g. tabulating the study intervention characteristics and comparing against the planned groups for each synthesis (item #5)). | 9-10 |
|  | 13b | Describe any methods required to prepare the data for presentation or synthesis, such as handling of missing summary statistics, or data conversions. | 9 |
|  | 13c | Describe any methods used to tabulate or visually display results of individual studies and syntheses. | 10 |
|  | 13d | Describe any methods used to synthesize results and provide a rationale for the choice(s). If meta-analysis was performed, describe the model(s), method(s) to identify the presence and extent of statistical heterogeneity, and software package(s) used. | 9-10 |
|  | 13e | Describe any methods used to explore possible causes of heterogeneity among study results (e.g. subgroup analysis, meta-regression). | 9-10 |
|  | 13f | Describe any sensitivity analyses conducted to assess robustness of the synthesized results. | 10 |
| Reporting bias assessment | 14 | Describe any methods used to assess risk of bias due to missing results in a synthesis (arising from reporting biases). | 10 |
| Certainty assessment | 15 | Describe any methods used to assess certainty (or confidence) in the body of evidence for an outcome. | 9 |
| **RESULTS** | | |  |
| Study selection | 16a | Describe the results of the search and selection process, from the number of records identified in the search to the number of studies included in the review, ideally using a flow diagram. | 11 |
|  | 16b | Cite studies that might appear to meet the inclusion criteria, but which were excluded, and explain why they were excluded. | 11 |
| Study characteristics | 17 | Cite each included study and present its characteristics. | 11 |
| Risk of bias in studies | 18 | Present assessments of risk of bias for each included study. | 11 |
| Results of individual studies | 19 | For all outcomes, present, for each study: (a) summary statistics for each group (where appropriate) and (b) an effect estimate and its precision (e.g. confidence/credible interval), ideally using structured tables or plots. | 11 |
| Results of syntheses | 20a | For each synthesis, briefly summarise the characteristics and risk of bias among contributing studies. | 11-14 |
|  | 20b | Present results of all statistical syntheses conducted. If meta-analysis was done, present for each the summary estimate and its precision (e.g. confidence/credible interval) and measures of statistical heterogeneity. If comparing groups, describe the direction of the effect. | 11-14 |
|  | 20c | Present results of all investigations of possible causes of heterogeneity among study results. | 11-14 |
|  | 20d | Present results of all sensitivity analyses conducted to assess the robustness of the synthesized results. | 14 |
| Reporting biases | 21 | Present assessments of risk of bias due to missing results (arising from reporting biases) for each synthesis assessed. | 14 |
| Certainty of evidence | 22 | Present assessments of certainty (or confidence) in the body of evidence for each outcome assessed. | 15 |
| **DISCUSSION** | | |  |
| Discussion | 23a | Provide a general interpretation of the results in the context of other evidence. | 16-19 |
|  | 23b | Discuss any limitations of the evidence included in the review. | 19 |
|  | 23c | Discuss any limitations of the review processes used. | 19 |
|  | 23d | Discuss implications of the results for practice, policy, and future research. | 20 |
| **OTHER INFORMATION** | | |  |
| Registration and protocol | 24a | Provide registration information for the review, including register name and registration number, or state that the review was not registered. | 6 |
|  | 24b | Indicate where the review protocol can be accessed, or state that a protocol was not prepared. | 6 |
|  | 24c | Describe and explain any amendments to information provided at registration or in the protocol. | 6, 9-10 |
| Support | 25 | Describe sources of financial or non-financial support for the review, and the role of the funders or sponsors in the review. | 21 |
| Competing interests | 26 | Declare any competing interests of review authors. | 21 |
| Availability of data, code and other materials | 27 | Report which of the following are publicly available and where they can be found: template data collection forms; data extracted from included studies; data used for all analyses; analytic code; any other materials used in the review. | 21-22 |

| **Supplementary Material 2 Detailed search strategy in four databases** | |
| --- | --- |
| Database | Search strategy |
| PubMed | ((("Terlipressin"[Mesh]) OR ((((((((Glycylpressin) OR (TGLVP)) OR (Terlypressin)) OR (Triglycyl Lysine Vasopressin)) OR (Triglycylvasopressin)) OR (Glipressin)) OR (Glypressin)) OR (Remestyp))) AND (("Surgical Procedures, Operative"[Mesh]) OR ((((((((((((((((Operative Procedures) OR (Operative Procedure)) OR (Procedure, Operative)) OR (Procedures, Operative)) OR (Surgical Procedure, Operative)) OR (Operative Surgical Procedures)) OR (Procedure, Operative Surgical)) OR (Procedures, Operative Surgical)) OR (Surgical Procedures)) OR (Procedure, Surgical)) OR (Procedures, Surgical)) OR (Surgical Procedure)) OR (Operative Surgical Procedure)) OR (Surgery, Ghost)) OR (Ghost Surgery)) OR (Surgery)))) AND (("Liver"[Mesh]) OR ((livers) OR (hepatic))) |
| EMBASE and CENTRAL ^a^ | 1 (Terlipressin or Glycylpressin or TGLVP or Terlypressin or Triglycyl Lysine Vasopressin or Triglycylvasopressin or Glipressin or Glypressin or Remestyp).af.  2 (Surgical Procedures, Operative or Operative Procedures or Operative Procedure or Procedure, Operative or Procedures, Operative or Surgical Procedure, Operative or Operative Surgical Procedures or Procedure, Operative Surgical or Procedures, Operative Surgical or Surgical Procedures or Procedure, Surgical or Procedures, Surgical or Surgical Procedure or Operative Surgical Procedure or Surgery, Ghost or Ghost Surgery or Surgery).af.  3 (Liver or livers or hepatic).af.  4 1 and 2 and 3 |
| Web of Science | 1 Terlipressin (Topic) or Glycylpressin (Topic) or TGLVP (Topic) or Terlypressin (Topic) or Triglycyl Lysine Vasopressin (Topic) or Triglycylvasopressin (Topic) or Glipressin (Topic) or Glypressin (Topic) or Remestyp (Topic)  2 Surgical Procedures, Operative (Topic) or Operative Procedures (Topic) or Operative Procedure (Topic) or Procedure, Operative (Topic) or Procedures, Operative (Topic) or Surgical Procedure, Operative (Topic) or Operative Surgical Procedures (Topic) or Procedure, Operative Surgical (Topic) or Procedures, Operative Surgical (Topic) or Surgical Procedures (Topic) or Procedure, Surgical (Topic) or Procedures, Surgical (Topic) or Surgical Procedure (Topic) or Operative Surgical Procedure (Topic) or Surgery, Ghost (Topic) or Ghost Surgery (Topic) or Surgery (Topic)  3 Liver (Topic) or livers (Topic) or hepatic (Topic)  4 ((#1) AND #2) AND #3 |
| CENTRAL, cochrane central register of controlled trials; ^a^ We retrieved articles from EMBASE and CENTRAL via the Ovid (https://ovidsp.ovid.com/). | |

| **Supplementary Material 3** **Definition of outcomes in included studies** | |
| --- | --- |
| Outcomes | Definition |
| Blood loss | Blood loss was recorded at the end of the surgical procedure by taking into account surgical sponges, suction canisters and the cell salvage device (if used). |
| Blood products transfusion | Packed red blood cells were transfused with a target hematocrit of 25% or 30% and hemoglobin of 8 g/dL or 7 g/dL. Other blood products were administered under guidance of rotational thromboelastometry. |
| AKI | **AKIN criteria:** **Stage I**, increase in sCr by >0.3 mg/dL or an increase to 150% to 200% from baseline (postoperative creatinine level divided by last creatinine level); **Stage II**, increase in sCr to 200% to 300% from baseline; and **Stage III**, increase in sCr to >300% from baseline or sCr >4 mg/dL, or treatment with new renal replacement therapy.  **KIDGO criteria:** AKI is defined as any of the following:(1) increase in sCr by ≥0.3mg/dL (≥26.5 μmol/L) within 48 hours; or (2) increase in sCr to ≥1.5 times baseline, which is known or presumed to have occurred within the prior 7 days; or (3) urine volume <0.5mL/kg/h for 6 hours.  **RIFLE criteria:** (within 7days) **Risk**, increase in sCr ×1.5 or GFR decrease >25%, urine volume less than 0.5 mL/kg/h for >6 hours; **Injury**, sCr ×2 or GFR decrease >50%, urine volume less than 0.5 mL/kg/h for >12 hours; **Failure**, sCr ×3, or sCr >4 mg/dL (354 mmol/L) with an acute rise >0.5 mg/dL (44 mmol/L) or GFR decrease >75%, urine volume less than 0.3 mL/kg/h for 24 hours or anuria for 12 hours; **Loss**, persistent acute renal failure: complete loss of kidney function >4 weeks; **End-stage kidney disease**, end-stage kidney disease >3 months. |
| Severe bradycardia | Heart rate below 50 beats per minute, needing pharmacological intervention and/or withdrawal of trial medication. |
| New-onset hypertension crisis | Diastolic readings greater than 90 mmHg needing the addition of antihypertensive medications, in a previously nonhypertensive patient after excluding other causes including inadequate analgesia and anxiety. |
| AKI, acute kidney injury; sCr, serum creatinine; GFR, glomerular filtration rate. | |

**Supplementary Material 4 Quality evaluation of the eligible RCTs with RoB2**

|  | Bias arising from the randomisation process | Bias due to deviations from intended interventions | Bias due to missing outcome data | Bias in measurement of the outcome | Bias in selection of the reported result | Overall bias |
| --- | --- | --- | --- | --- | --- | --- |
| Abbas, 2019 | + | + | + | + | + | + |
| Fayed, 2013 | ? | ? | + | + | + | ? |
| Hashim, 2023 | - | - | + | + | + | - |
| Hong, 2012 | ? | - | + | + | + | - |
| Ibrahim, 2015 | + | ? | + | ? | + | ? |
| Kohler, 2019 | + | + | + | + | + | + |
| Mahdy, 2019 | + | + | + | + | + | + |
| Mukhtar, 2011 | + | ? | + | + | + | ? |
| Reddy, 2017 | + | + | + | + | + | + |
| Zhang, 2023 | + | + | + | + | + | + |

| + | Low risk of bias |
| --- | --- |
| ? | Some concerns |
| - | High risk of bias |

RCTs, randomized controlled trials; RoB2, version 2 of the Cochrane risk-of-bias tool.

| **Supplementary Material 5 Quality evaluation of the eligible case-control studies with NOS** | | | | | | | | | |
| --- | --- | --- | --- | --- | --- | --- | --- | --- | --- |
| Study | Selection | | | | Comparability | | Outcome | | |
|  | Representative-ness | Selection of  non-exposed | Ascertainment  of exposure | Outcome not present at start | Comparability on most important factors | Comparability on other risk factors | Assessment of outcome | Long enough follow-up (median≥7 days) | Adequacy  (completeness) of follow-up |
| Karaaslan, 2018 | * | * | * | * | - | - | * | - | * |
| Mukhtar, 2015 | * | * | * | * | * | - | * | - | * |
| NOS, Newcastle–Ottawa scale; *indicates criterion met; - indicates significant of criterion not met. | | | | | | | | | |

| **Supplementary Material 6** **Demographics and clinical characteristics of included studies** | | | | | | | | | |
| --- | --- | --- | --- | --- | --- | --- | --- | --- | --- |
| Outcomes | Studies | No. of patients | WMD or RR | 95%CI | *P*-value | Heterogeneity | | | |
|  |  | TP/without TP |  |  |  | Chi^2^ | df | *P*-value | I^2^(%) |
| Age(years) | 11 | 359/347 | 0.96 | [-1.05,2.96] | 0.35 | 23.63 | 10 | 0.009 | 58 |
| Gender(female) | 10 | 385/488 | 0.93 | [0.73,1.18] | 0.54 | 4.57 | 9 | 0.87 | 0 |
| BMI(kg/m^2^) | 7 | 189/178 | 0.57 | [0.03,1.12] | 0.04^a^ | 6.07 | 6 | 0.42 | 1 |
| MELD score | 8 | 217/205 | 0.19 | [-0.38,0.77] | 0.51 | 9.21 | 7 | 0.24 | 24 |
| Preoperative AST (IU/L) | 5 | 220/209 | -0.45 | [-4.77,3.87] | 0.84 | 3.43 | 4 | 0.49 | 0 |
| Preoperative ALT (IU/L) | 5 | 220/209 | 1.38 | [-4.40,7.16] | 0.64 | 2.07 | 4 | 0.72 | 0 |
| Preoperative sCr (IU/L) | 8 | 248/237 | 0.03 | [-0.02,0.08] | 0.22 | 14.20 | 7 | 0.05 | 51 |
| BMI, body mass index; MELD, model of end liver disease; AST, aspartate aminotransferase; ALT, alanine aminotransferase; sCr, serum creatinine; TP, terlipressin; WMD, weighted mean difference; RR, risk ratio; CI, confidence interval.  ^a^ Statistically significant. | | | | | | | | | |

**Supplementary Material 7 Forest plot of red blood cell transfusion between the TP group and the Control group**

TP, terlipressin; SD, standard deviation; IV, inverse variance; CI, confidence interval.

**Supplementary Material 8 Forest plot of fresh frozen plasma between the TP group and the Control group**

TP, terlipressin; SD, standard deviation; IV, inverse variance; CI, confidence interval.

**Supplementary Material 9 Forest plot of intraoperative urine output between the TP group and the Control group**

TP, terlipressin; SD, standard deviation; IV, inverse variance; CI, confidence interval.

**Supplementary Material 10 Forest plot of postoperative serum creatine between the TP group and the Control group**

(a) peak serum creatine; (b) serum creatine at last follow-up. TP, terlipressin; SD, standard deviation; IV, inverse variance; CI, confidence interval.

**Supplementary Material 11 Forest plot of postoperative hepatic function between the TP group and the Control group**

(a) peak aspartate aminotransferase; (b) aspartate aminotransferase at last follow-up; (c) peak alanine aminotransferase; (d) alanine aminotransferase at last follow-up. TP, terlipressin; SD, standard deviation; IV, inverse variance; CI, confidence interval.

**Supplementary Material 12 Forest plot of TP-related adverse events between the TP group and the Control group**

(a) severe bradycardia; (b) new-onset hypertension crisis; (c) peripheral skin changes. TP, terlipressin; M-H, Mantel-Haenszel; CI, confidence interval.

**Supplementary Material 13 Forest plot of operating time between the TP group and the Control group**

TP, terlipressin; SD, standard deviation; IV, inverse variance; CI, confidence interval.

**Supplementary Material 14 Forest plot of intensive care unit stay between the TP group and the Control group**

TP, terlipressin; SD, standard deviation; IV, inverse variance; CI, confidence interval.

**Supplementary Material 15 Forest plot of postoperative complications between the TP group and the Control group**

(a) pneumonia; (b) reoperation; (c) biliary complications; (d) vascular complications; (e) intra-abdominal haematoma; (f) rejection.

TP, terlipressin; M-H, Mantel-Haenszel; CI, confidence interval.

**Supplementary Material 16 Sensitivity analysis**

(a) estimated blood loss; (b) red blood cell transfusion.

**Supplementary Material 17 Egger's regression tests**

(a) estimated blood loss; (b) red blood cell transfusion; (c) fresh frozen plasma; (d) acute kidney injury rate; (e) intraoperative urine output; (f) peak serum creatine; (g) serum creatine at last follow-up; (h) peak aspartate aminotransferase; (i) aspartate aminotransferase at last follow-up; (j) peak alanine aminotransferase; (k) alanine aminotransferase at last follow-up; (l) length of stay; (m) operating time; (n) intensive care unit stay; (o) pneumonia;

(p) reoperation; (q) biliary complications; (r) vascular complications.
